# An extensive description of the microbiological effects of silver diamine fluoride on dental biofilms using an oral in situ model

**DOI:** 10.1038/s41598-022-11477-1

**Published:** 2022-05-06

**Authors:** Kittipit Klanliang, Yoko Asahi, Hazuki Maezono, Maki Sotozono, Nanako Kuriki, Hiroyuki Machi, Shigeyuki Ebisu, Mikako Hayashi

**Affiliations:** 1 Department of Restorative Dentistry and Endodontology, Osaka University Graduate School of Dentistry, 1-8 Yamadaoka, Suita, Osaka 565-0871 Japan; 2 Division of Cariology, Operative Dentistry, and Endodontics, Department of Oral Health Science, Niigata University Graduate School of Medical and Dental Sciences, Niigata, 951- 8514 Japan; 3 Osaka University Dental Technology Institute, 1-8 Yamadaoka, Suita, Osaka 565-0871 Japan

**Keywords:** Microbiology, Dentistry

## Abstract

Silver diamine fluoride (SDF) has been long studied in laboratories, and its clinical effectiveness in the treatment and prevention of root caries has been reported. In the present study, we assessed the microbiological effects of SDF on dental biofilms grown on demineralized dentin in situ. Specifically, demineralized bovine root dentin slabs used as biofilm substrates were treated with 38% SDF, and the biofilms formed after this treatment were analyzed via real-time PCR, DEAD/LIVE cell staining, and SEM. Next, the viable cell count was determined, and microbial profiles were compared using 16S rRNA gene sequencing. Untreated slabs were used as controls. We observed significant decreases in viable cell counts (p < 0.05), number of biofilm-forming cells (p < 0.01), biofilm thickness (p < 0.01), and high proportion of dead cells with SDF treatment (p < 0.01). The microcolonies in the SDF-treated biofilms showed less complexity, and only a limited number of genera were differentially abundant between the groups. Microbial diversity index comparisons showed no significant differences between the groups with respect to treatments days (p = 0.362). Thus, SDF negatively influenced dental biofilm growth on demineralized root dentin in situ; however, its antimicrobial action did not target a specific oral taxon.

## Introduction

Oral biofilms are polymicrobial communities adhered to oral cavity surfaces and consist of multiple species of microorganisms embedded within a matrix containing extracellular polysaccharides. It is generally accepted that the interactions within and among oral biofilms, as well as with the host, are potentially responsible for oral health and disease states^1–3^. Dental caries is a prime example of oral infectious diseases that originate as a consequence of the interactions between microorganisms, hosts, and environmental conditions^1^. The etiology of dental caries has presumably been described as a shift to the predominance of some cariogenic species within symbiotic biofilms that eventually leads to an imbalance between demineralization and remineralization of tooth structures^4–6^.

Human life expectancy has increased, leading to increased awareness of dental health, including advanced oral therapeutic modalities. The number of remaining teeth with gingival recession has become significantly greater in the elderly. Once root surface/dentin is exposed to the oral environment, it is likely to be susceptible to demineralization owing to the acidic environment created by acid-producing bacteria in the supragingival biofilm. Compared to coronal enamel, root dentin and cementum are more susceptible to acidic pH due to their lower inorganic content and smaller size of hydroxyapatite crystallites^7^. In the elderly, decreased salivary flow resulting from medications or systemic diseases and difficulty in brushing the teeth regularly due to physical impairment can increase the risk of root caries development^8,9^, which presently is a crucial oral problem affecting them and worsening their quality of life.

Silver diamine fluoride (SDF) is an aqueous therapeutic agent containing silver and fluoride that is used to arrest caries and promote remineralization. Its clinical efficiency in the treatment of coronal caries of primary^10,11^ and permanent dentitions^12,13^, including root surface caries^14^, has been reported. SDF solution is also used for the treatment of dentinal hypersensitivity^15^. Further, a number of in vitro studies have demonstrated its efficacy in reducing mineral loss^16,17^, enhancing dentin hardening^18^, and ensuring collagen preservation, as inferred from an experiment involving acid challenge^17^. Furthermore, SDF can react with the inorganic components of the tooth and form fluorohydroxyapatite, which can promote tooth hardness and decrease the solubility of the affected dentin^19^. Additionally, the antimicrobial properties of SDF remarkably inhibit root caries progression. Specifically, it has been demonstrated that the growth rate of a monoculture of *Streptococcus mutans* is significantly lower under SDF treatment than under silver fluoride and potassium iodide treatment^20^. Moreover, it has also been observed that biofilm formation by *Actinomyces naeslundii* on dentin blocks decreases significantly after the direct application of SDF^18^. This is consistent with the results of a more recent study in which dual- and multi-species biofilm models were used^21,22^.

At present, 16S rRNA gene sequencing in combination with microbiological ecology methods has become a crucial part of studying oral microorganisms in health and diseases^23^. A recent clinical sample study on the root caries-related microbial profiles observed before and after SDF application on cavitated lesions of the root surface showed that even though there was no statistical difference in microbial diversity after SDF application, the relative abundance of cariogenic taxa (e.g., *Scardovia*, *Bifidobacterium*, and *Actinomyces*) decreased with time^24^. Therefore, it is presumed that the antimicrobial properties of SDF, as exhibited in the oral microbiome, play an important role in the prevention of the recurrence of root caries. However, no clear evidence that accurately validates these assumptions, particularly in relation to actual dental biofilms, has been reported. Hence, a combination of comprehensive quantitative data acquisition and gene-based characterization of microorganisms in the biofilm formed on SDF-applied tooth surfaces would provide a better understanding of the antibacterial effects of SDF.

The advancement of in vitro biofilm models has mitigated the complexity in studying oral biofilm nowadays^25,26^. Nevertheless, there are some challenging aspects that limit the capability of in vitro models in mimicking the natural oral condition, including the species diversity and the complex environment of the oral cavity^26^. Therefore, an in situ biofilm model could be considered as an alternative for the investigation of natural oral biofilms and various therapeutic modalities. To extend the microbiological perspective of using SDF as a topical medication for arresting and preventing dental caries, we therefore assessed the microbiological effect of 38% SDF on dental biofilms grown on demineralized dentin in situ in terms of bacterial viability and biomass. Furthermore, based on 16S rRNA gene sequencing technique, we analyzed bacterial profiles and changes in biofilm biodiversity, to observe the changes in the dental biofilm community presumably influenced by treatment with SDF. The null hypotheses of this study were that (i) the application of SDF on demineralized dentin does not inhibit the growth and viability of biofilms and (ii) there is no difference in the microbial community structure between the SDF and control groups.

## Results

### Quantity of bacteria in biofilm

**Figure 1:**
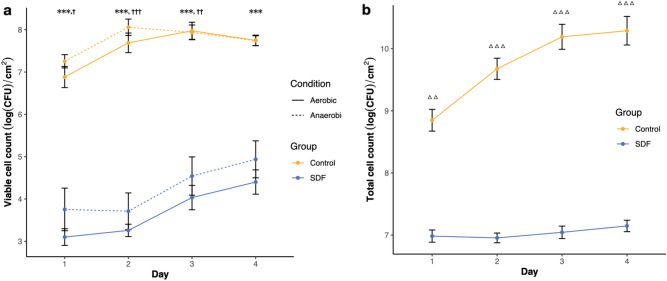
(**a**) Bacterial viability (log colony-forming unit (CFU) per unit area) under aerobic and anaerobic conditions. ***p < 0.001, aerobic condition; ^†^p < 0.05, ^††^p < 0.01, ^†††^p < 0.001, anaerobic condition. (**b**) Real-time PCR quantification of biofilm-forming cells (log CFU per unit area). ^△△^p < 0.01, ^△△△^p < 0.001.

Under both aerobic and anaerobic conditions, cultivable bacteria cell count revealed that the log CFU counts corresponding to the SDF-treated biofilms were significantly lower than those corresponding to the controls (Repeated Measure ANOVA, p < 0.01) (Fig. 1a). Consistent with the viable cell count, quantification via real-time PCR showed the same tendency, i.e., the SDF treatment group showed a lower bacterial load (Repeated Measure ANOVA, p < 0.001) (Fig. 1b).

### Biofilm biomass, thickness, and proportion of dead and live cells based on confocal laser scanning microscopy

**Figure 2:**
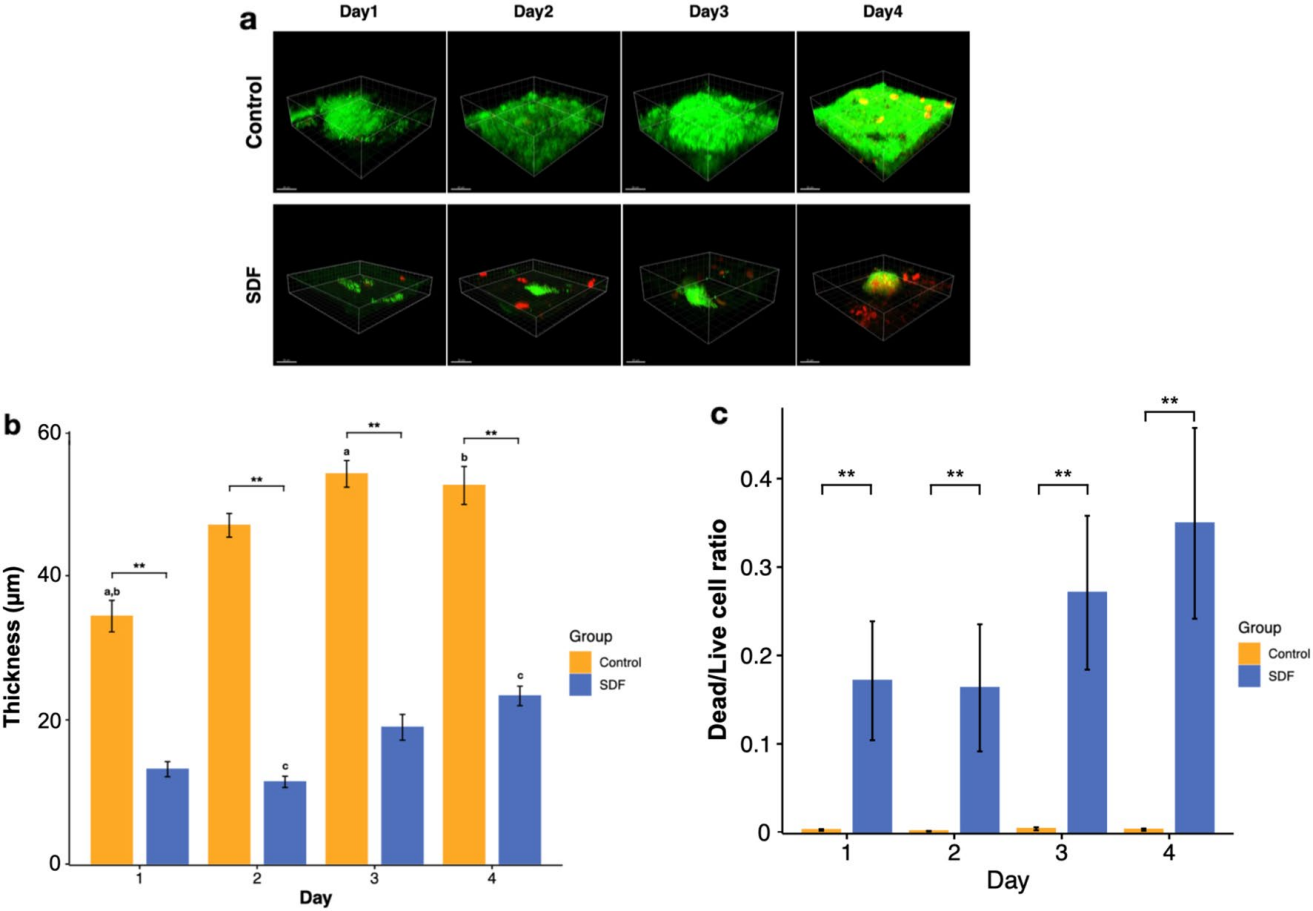
Confocal laser scanning microscopy (CLSM) analysis of biofilms grown on dentin slabs in the control and SDF groups using the Imaris imaging software. (**a**) Representative LIVE/DEAD staining images comparing biofilms formed in each group over the experiment days. Live and dead cells are labelled in green and red, respectively (scale bar, 20 μm). (**b**) Thickness of biofilms p** < 0.01. (**c**) Ratio of live to dead cells. p** < 0.01. The letters a, b, and c denote significant differences over different experiment days in the same group (p < 0.05).

The representative images corresponding to LIVE/DEAD bacterial cell staining obtained via confocal microscopy (Fig. 2a) revealed a noticeably lesser biofilm mass in the SDF group, with dead cells labelled in red enclosed within microcolonies. Although the biofilm biomass in both groups tended to increase with time, as demonstrated by the thickness of biofilm in Fig. 2b (p < 0.01), the dead to live cell volume ratio of the SDF-treated biofilm was significantly greater than that of the corresponding control group throughout the experimental period (p < 0.01) (Fig. 2c).

### Microscopic structure of biofilms based on SEM

**Figure 3:**
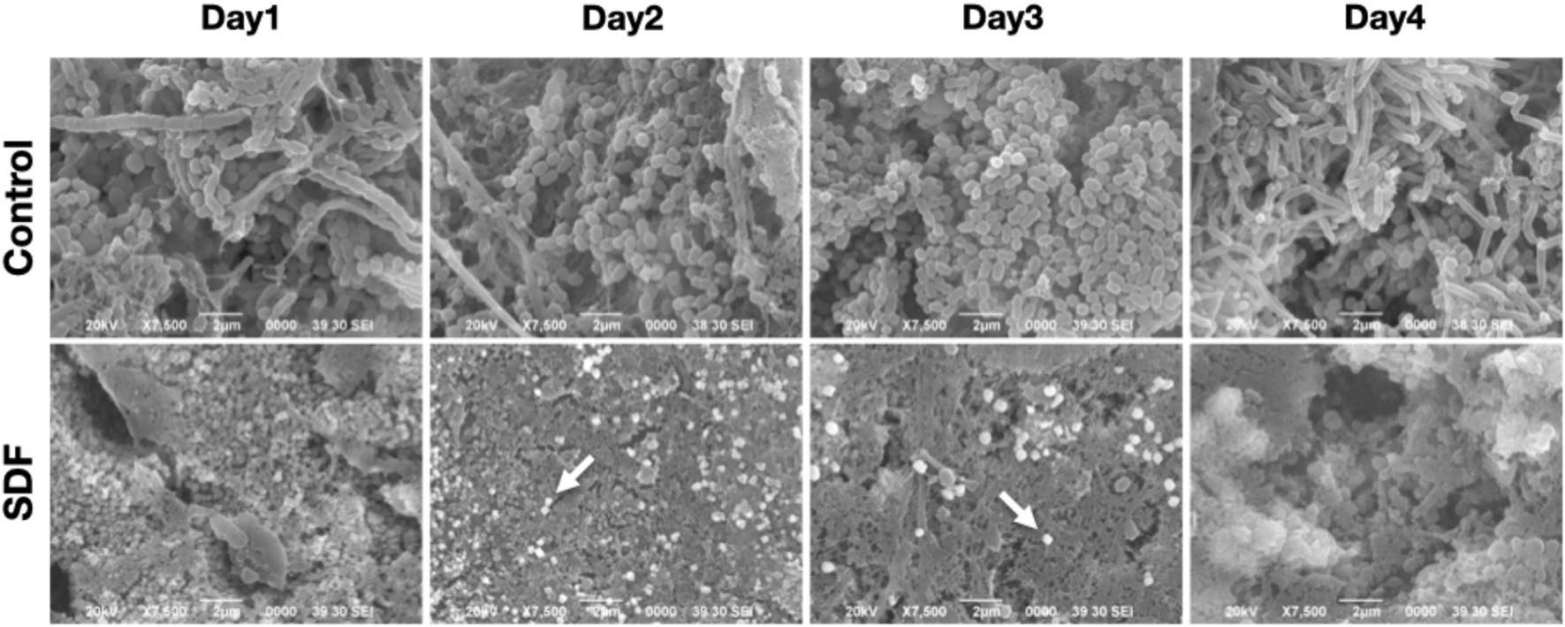
Scanning electron microscopy images of biofilms corresponding to the control and SDF groups based on experiment day (× 7500). White arrows indicate the silver particles that precipitated on the dentin slabs.

High-magnification SEM images were captured to observe the characteristics of the ultrastructure of in situ biofilms of both groups. Figure 3 shows the co-aggregation of rod-shaped and columnar microcolonies, which penetrated the dentinal tubules of bovine root dentin in the control group on the first day. Further, the biofilm structure became more complex owing to the presence of substantial filamentous and coccal bacteria that grew in the subsequent days. In contrast, the microcolonies in the biofilms corresponding to the SDF group were sparsely detectable and were less complex. We also observed that small silver particles precipitated on the dentin surface in the SDF group.

### Microbial profile based on 16S rRNA gene sequencing

With respect to the results of 16S rRNA gene sequencing involving a total of 80 samples (40 each from the control and SDF groups), 14 phyla, 21 classes, 33 orders, 58 families, and 90 genera were detected across all samples. Based on the relative abundances of the different taxa, the most abundant phyla in the control and SDF groups were *Proteobacteria* (31.54%) and *Firmicutes* (37.20%), respectively. Further, at the genus level, the three most abundant phyla in the control group were *Neisseria* (18.79%), *Streptococcus* (15.33%), and *Porphyromonas* (13.19%), while *Streptococcus* (22.17%), *Haemophilus* (11%), and *Neisseria* (10.96%) were the most abundant phyla in the SDF group. Furthermore, we examined the extent of changes in the relative abundances of the different genera. Table 1 shows the differential abundances of the taxa detected between the groups, compared on each experiment day. Among the more abundant taxa (average relative abundance > 3%), only *Actinomyces* (Log2FoldChange = − 2.68) was found to be significantly more abundant in the SDF group on day 3 (adjusted p-value < 0.001). However, *Porphyromonas* was in the control group (Log2FoldChange ranging from 1.36 to 2.42). Additionally, considering the pooled data between the groups, *Granulicatella* and unclassified genera in the family Gemellaceae were observed more frequently in the SDF treatment group.

**Table 1:** Significant genera with differential changes in relative abundance expressed as Log2FoldChanges at the genus level based on the comparison between pooled samples corresponding to the control and SDF groups and among the counterpart data corresponding to groups and days.

Bacterial taxa	Log2FoldChange	Adjusted p-value
**Overall**		
*Porphyromonas*	1.095	8.43479E−04
*Neisseria*	0.818	7.99910E−03
*Granulicatella*	− 1.079	8.01837E−03
*Family Gemellaceae*	− 1.026	1.11891E−02
**Day1**		
*Neisseria*	1.49	3.66211E−02
*Porphyromonas*	1.446	4.57301E−02
**Day2**		
*Porphyromonas*	2.419	9.67159E−08
*Neisseria*	1.553	2.83632E−04
*Actinobacillus*	2.639	5.16592E−03
**Day3**		
*Campylobacter*	2.431	5.43872E−06
*Actinomyces*	− 2.68	8.30128E−06
*Porphyromonas*	1.355	1.72108E−03
**Day4**		
*Campylobacter*	4.39	1.70780E−10
*Fusobacterium*	3.058	9.86462E−07
*Prevotella*	2.851	9.44494E−06
*Capnocytophaga*	2.52	2.78876E−03
*Neisseria*	1.861	1.93780E−02
*Porphyromonas*	2.034	1.56524E−04
*Rothia*	2.412	6.53380E−05
*Veillonella*	2.279	5.97859E−03
*Haemophillus*	1.398	4.04648E−02

### Bacterial diversity of biofilm community

**Figure 4:**
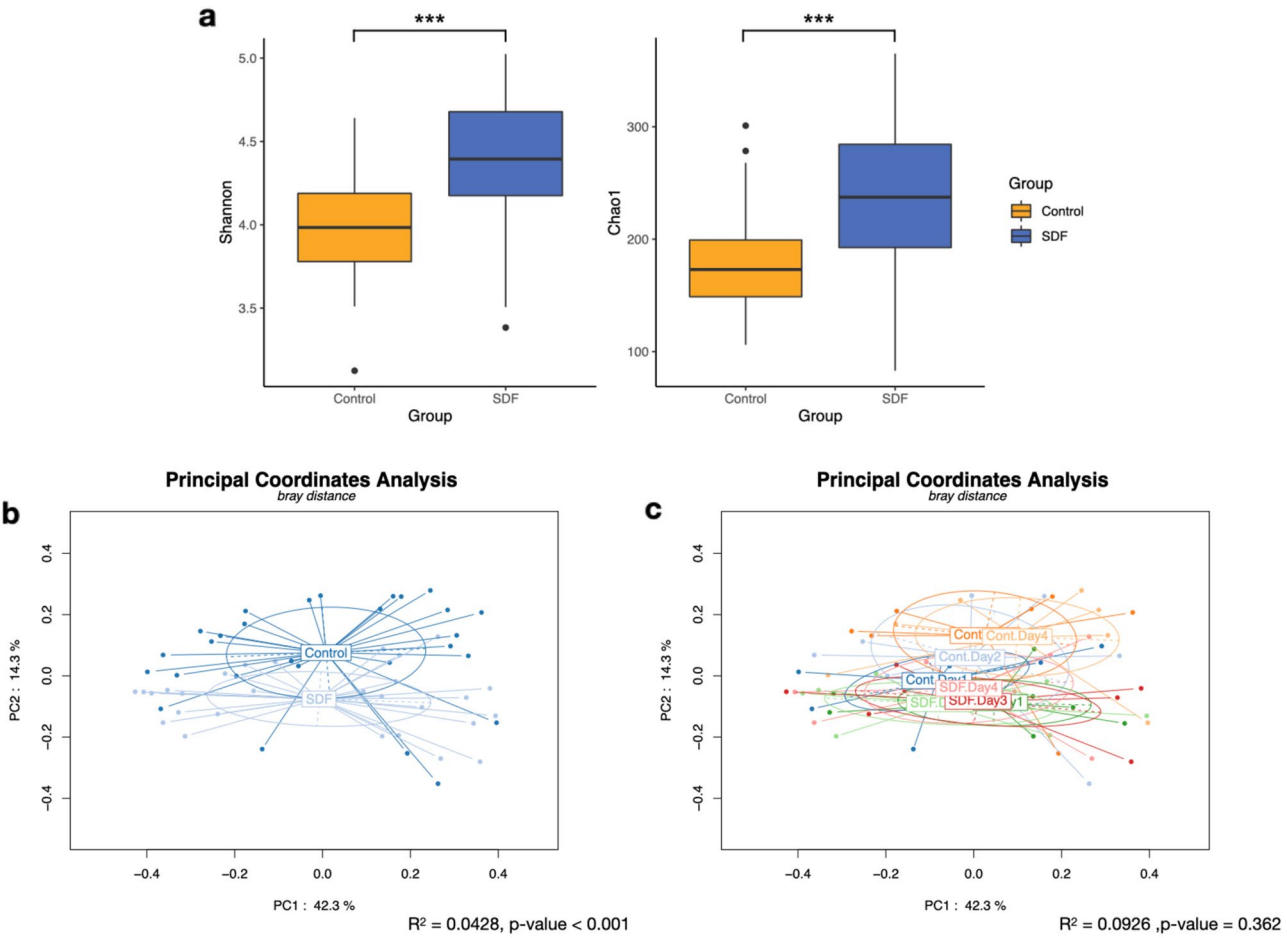
Alpha and beta diversities of the bacterial community on biofilms. (**a**) Shannon and Chao1 diversity indices. (**b**) Principal coordinates analysis (PCoA) plot for pooled biofilm samples from the control and SDF groups based on Bray–Curtis distance. (**c**) PCoA plot based on Bray–Curtis distance when group and day factors were considered together. Statistical comparisons were done using the PERMANOVA test.

To further assess the effect of SDF application on community structure, alpha diversity parameters, including Chao1 and Shannon indices, were calculated. The calculated Chao1 indices showed significant differences between the control and SDF groups, regardless of the experiment day (control, 183.25 ± 46.82; SDF, 235.03 ± 65.09; adjusted p-value < 0.001). Similarly, the two groups also showed significant differences in Shannon indices (control, 3.99 ± 0.33; SDF, 4.38 ± 0.40; adjusted p-value < 0.001) (Fig. 4a). In contrast, when both indices were compared based on the experiment day, no significant differences were observed, except on day 2 for Shannon index. The PERMANOVA test showed significant differences in beta-diversity based on the Bray–Curtis distances between the biofilm communities when the groups were compared (adjusted p-value < 0.001). However, groups showed no significant differences with respect to the experiment day. Further, the PCoA plots did not show any clear clustering pattern for any of the factors considered (Fig. 4b,c).

## Discussion

In this study, we extensively assessed the effect of SDF treatment on dental biofilms from a microbiological perspective, combining conventional cultivable and microbial community profile analyses using current gene sequencing methods. Our first null hypothesis was rejected based on the results as a remarkable biofilm-inhibitory effect was observed with SDF treatment. In contrast, our findings supported the second null hypothesis, as the results did not demonstrate significant differences in microbial diversity between the biofilms of SDF and control groups on any experiment day.

To overcome the limitations associated with in vitro dental biofilms, we used a newly designed dentin slab-holding oral device to enable the growth of dental biofilms under actual oral conditions. Reportedly, bovine root dentin, which was used as a substrate in our in situ model instead of human root dentin, is suitable for investigating anticariogenic agents^27^, although its microhardness was found to be lower in another in situ study^28^. Furthermore, bovine dentin being more readily available and easy to manipulate than human dentin makes it more suitable for studies like ours, in which a large number of dentin slabs are required^29^. However, using sodium hypochlorite for dissolving the remaining organic tissues on root dentin after preparation might alter its surface characteristics, affecting the ability of the substrate to completely mimic the actual oral conditions. Hence, the results should be interpreted with caution.

Dental caries is caused by the acidic environment created by acidogenic and aciduric bacteria that leads to an imbalance between demineralization and remineralization on the tooth surface^7^. Carious lesions on the root surface are initiated by a polymicrobial biofilm covering the root surface and its metabolic products. The microbiota of root caries has been extensively studied, using both culture-based^30–32^ and non-culture-based^33,34^ approaches, and reportedly, it includes a variety of related bacterial species. Moreover, biodental engineering factors, such as chewing and parafunctional habits, capable of generating electrochemical transfer from the tooth surface to saliva and eventually leading to surface demineralization are involved in root caries etiology^35^. It has been reported that exposed root surface is a risk indicator associated with root caries in the elderly^36^. Traumatic tooth brushing is an etiological factor for gingival recession that exposes the root surface^37^. Consequently, improper brushing might increase the risk of root caries in older people.

For several decades, SDF has been used to prevent and arrest carious lesions in primary teeth^11,38^ and the root surface, particularly in the elderly^13^. However, the specific mechanism by which it exerts this effect has not yet been fully elucidated. Several in vitro studies have been conducted to investigate the underlying mechanisms extensively. Apart from its efficacy in preventing the degradation of dentin collagen^17^ and increasing microhardness as well as mineral contents after application^18,39^, it has also been demonstrated that SDF exerts various effects on cariogenic bacteria. Silver ions bind strongly to sulfhydryl groups and proteins on bacterial cell membranes, inhibiting intracellular enzyme activity as well as DNA replication^40^, eventually causing bacterial cell death and inhibiting biofilm formation^41^. A monoculture study of *S. mutans* and *A. naeslundii* showed significantly fewer CFU counts in the SDF treatment group than in the control^18^. This is also consistent with the results of another study that was performed using a co-culture model of *S. mutans* and *Lactobacillus acidophilus* on demineralized dentin blocks, which showed significantly lower CFU counts in SDF-treated blocks than water-applied blocks^22^. Similarly, the application of 38% SDF on cariogenic biofilms consisting of *S. mutans*, *S. sorbrinus*, *L. acidophilus*, *Lactobacillus rhamnosus,* and *A. naeslundii*, could inhibit the growth of this mixed-species biofilm^21^. Data from our present study on viable cell counts are consistent with the results of these aforementioned studies, demonstrating decreased CFU in the SDF group under both aerobic and anaerobic culture conditions. Similarly, real-time PCR quantification revealed a significantly lower total number of bacterial cells in the SDF group than in the control group, indicating that SDF reduces viable cell count in the biofilms formed on dentin slabs. Further, the viable cell count corresponding to the control group from both analyses exhibited a similar trend as that observed in a previous in situ study using hydroxyapatite (HA) disks^42^, suggesting the potential for the application of HA disks as a practical alternative to the bovine root dentin substrate for dental biofilm cultivation.

Regarding the biomass of the dental biofilms, CLSM images showed noticeably lesser biofilm formation, with dead cells, on the SDF-treated dentin slabs than in the control group. These findings are in accordance with previous in vitro studies^18,21,22^, although the SDF application sequences were different, and our experimental period was shorter. Similarly, the lower thickness of the biofilms following SDF treatment and their lower bacterial cell viability, as demonstrated by the dead to live cell ratio, indicated the anti-biofilm efficacy of SDF after application on demineralized root dentin. The CLSM results were further corroborated by the ultrastructural characterization of the biofilms based on SEM. While the actual maturation of biofilms could be observed in the control group as previously demonstrated in vivo^43^ and in situ using HA disks^42^, which showed the co-aggregation of various bacteria and the presence of matrix-like structures after 48 h of experimentation, biofilms on the dentin surfaces treated with SDF were scarce, and the bacterial composition was less complex. Additionally, consistent with earlier in vitro studies^18,21^, precipitated silver particles were also observed on the SDF-treated dentin surfaces in the present study. It has also been suggested that silver and fluoride ions in SDF interact with HA in the tooth and subsequently form CaF_2_ and Ag_3_PO_4_^44^. In particular, CaF_2_ is a crucial fluoride reservoir that can eventually react with HA and form fluoroapatite, which reportedly is a favorable acid-resistant crystalline matrix that reduces the susceptibility of the tooth surface to demineralization^10,19,45^. Additionally, Ag_3_PO_4_ reacts with alkali chlorides to form AgCl, which is the major precipitate found on SDF-treated surfaces^17,19^, and owing to its low solubility, it has been affirmed that it has a slow-release effect with respect to silver ions that is responsible for its antibacterial properties^46^. Furthermore, it has been considered that the pronounced antibacterial effect of SDF is derived from the metallic silver nanoparticles that are also produced following the reaction between SDF and HA^17^. These particles are inert, but after coming in contact with moisture in the oral cavity, they release silver ions^47^. In a recent study, it was demonstrated that SDF-coated root dentin significantly attenuates lactate production in *S. mutans*^48^. For these reasons, SDF is considered an efficient agent for arresting caries.

In this study, biodiversity analyses using 16 s rRNA sequencing were performed to investigate the changes in the biofilm community on root dentin following SDF application. The alpha diversity indices (both Shannon and Chao1 indices) corresponding to the SDF group when data were pooled together regardless of the experiment day, were significantly greater than those corresponding to the control, suggesting that the bacterial taxa detected in the SDF group were richer and more equally distributed than those in the control group. However, most of the comparisons with respect to the experiment day showed no significant differences between the groups. These results are consistent with those of previous studies on gene sequencing in plaques, in which the effect of SDF application on bacterial profiles was investigated using plaque samples^24,49^. Moreover, performing the PERMANOVA test of Bray–Curtis distances revealed that SDF application had a significant effect on the bacterial communities between the groups, within the groups, and with respect to time. The PERMANOVA test also showed that the community structure corresponding to the SDF-treated biofilms was different from that corresponding to the control biofilms; this is inconsistent with the results obtained in a previous study^24^. This could be attributed to the differences in the experimental design; the method for normalization, including the statistical analyses conducted^50^; delayed colonization, which possibly influenced the number of bacterial populations observed in the community analysis; and the type of bacterial taxa in the initial biofilm.

Our differential abundance analysis revealed only four significant differences among the most frequently encountered taxa when collective data between groups were compared. *Granulicatella* and an unclassified genus in the *Gemellaceae* family were frequently detected in the SDF biofilms, whereas *Porphyromonas* and *Neisseria* were found to be significantly subtle. Further, *Granulicatella* and *Gemella* species are gram-positive facultative anaerobes, whereas *Neisseria* is gram-negative and predominantly colonizes aerobic environments. These microbes are considered commensal among oral flora and are frequently observed at all sites of the oral mucosal tissue^51–53^. Furthermore, oral *Porphyromonas*, such as *P. gingivalis,* is a gram-negative anaerobe that plays a key role in many oral infections, including periodontitis^54^. The results of this study possibly indicate a non-specific effect of SDF on the oral microbiome, given that changes in the relative abundances of both commensal and pathogenic bacteria were observed. *Actinomyces* were more abundant in the SDF group; however, based on the experiment day we observed that significant abundance could be detected only on days 2 and 3. This is consistent with the findings of Mei et al., who demonstrated that *Actinomyces* numbers tended to increase in arrested coronal caries in children after treatment with SDF^49^.

An evidence-based clinical practice guideline has been proposed by the American Dental Association for Evidence-Based Dentistry. According to it, biannual application of SDF is recommended to arrest advanced cavitated carious lesions on coronal surfaces, based on the highest rate of caries arrest observed in a clinical trial. It is also recommended to use SDF annually to arrest non-cavitated or cavitated root caries lesions^55^. Though we could not find guidelines for clinical use to prevent the onset of root caries by applying SDF to the exposed root surfaces, it has been reported that applying SDF on the exposed root surfaces may reduce the initiation of root caries^56^. This is also supported by the present study that shows a significant reduction in biofilm formation on demineralized root dentin in situ.

One of the limitations of this study is the short experimental period. The cultivation of biofilms was performed within four consecutive days of the start of the study owing to the practical difficulties associated with the availability of volunteers agreeing to wear the device along with their concerns regarding oral hygiene. Furthermore, since our 16S rRNA gene sequence analysis did not identify microbiota at the species level, our findings are restricted to the genus level, which can be elusive; thus, there is a necessity for careful consideration of the microbial abundance data interpretation. In conclusion, the application of SDF reduced the growth of dental biofilms grown on demineralized dentin in terms of bacterial cell viability and biofilm biomass under in situ conditions. Furthermore, even though SDF did not clearly alter the microbiome diversity and the differential abundances of specific bacterial genera, its potential antimicrobial effect is still recognized as a significant mode of action for root caries therapeutic interventions. Furthermore, its substantivity on tooth structures, particularly on the root dentin surface, would be valuable for future investigations, with a focus on establishing a protocol for SDF therapy and estimating the appropriate interval for re-application.

## Conclusion

The application of SDF solution on demineralized dentin negatively affected the growth of dental biofilm by reducing bacterial cell viability and total biomass. However, bacterial composition analysis revealed that the antimicrobial effect of SDF did not directly influence the community structure of the dental biofilm. The efficiency of SDF in preventing and treating root caries may potentially arise from the biofilm inhibitory effect rather than driving the change in bacterial composition.

## Methods

### Volunteer recruitment

The study design, which included the usage of bovine dentin slabs inside the volunteer’s mouth, was approved by the Ethics Committee of the Osaka University Graduate School of Dentistry (approval number: H29-E17-2, approval date: 2017/9/27) and was conducted according to the guidelines of the Declaration of Helsinki. Ten healthy volunteers (students and staff of the Osaka University Graduate School of Dentistry), aged 26 to 31 years, were recruited for this study. The inclusion criteria were as follows: (1) no clinical signs of caries, gingivitis, or periodontitis, (2) no history of antibiotics usage for 3 months, (3) no history of smoking, and (4) no history of orthodontic treatment and denture use. All the volunteers were informed about the study protocols before signing an informed consent form.

### Dentin slab preparation and the in situ model

The customized in situ device used in this study was a modification of the biofilm model developed in previous studies^42,57^. Briefly, each intraoral device, which had left and right pieces, was designed to have eight rectangular slots for positioning the substrate used for biofilm cultivation. The slots were perforated to create a 1 mm × 3 mm opening at the buccal surface using a round diamond bur (Supplementary Fig. S1). Further, the root dentin of bovine anterior incisors was prepared for the study by cutting them into rectangular slabs. First, the bovine roots were cut along the first plane (thickness) using a cutting machine (IsoMet™; Buehler, Lake Bluff, IL, USA). Thereafter, the pieces of the dentin were further cut along the second plane (width and length) to match the designated dimensions (3 × 5 × 1 mm^3^) using a diamond wire saw (Well Diamond Wire Saws; Norcross, GA, USA). The remaining organic tissues in the dentin slabs were dissolved by ultrasonication with an aqueous solution of 2.5% sodium hypochlorite for 60 min (replenished every 10 min) prior to demineralizing the surface via ultrasonication with 20% citric acid for 30 min. The prepared dentin slabs were assigned to the control and SDF groups. The slabs assigned to the SDF group were treated with 38% SDF for 4 min before washing with distilled water for 30 s. Subsequently, the slabs were fit in the intraoral devices and sterilized using ethylene oxide gas (Supplementary Fig. S2).

### Experimental design

The study volunteers were asked to wear the left and right pieces of the devices, each of which contained eight dentin slabs, for a total of 96 h to enable biofilm growth. Four dentin slabs with biofilms were extracted at 24, 48, 72, and 96 h, respectively, from both groups and subjected to different biofilm analyses. Throughout the experiment, all the sample collection procedures were performed at the same period for all the volunteers, who were allowed to remove the devices only during meals and when drinking. Tooth brushing was allowed but without toothpaste.

### Viable cell count

After extraction, the dentin slabs were gently washed twice, vortexed for 30 s, and ultrasonicated in distilled water at 4 °C for 15 min to detach the biofilms. The resulting bacterial suspension was serially diluted by tenfold. Thereafter, droplets from each dilution were cultured on Columbia blood agar plates with 5% sheep blood (Nippon Becton Dickinson, Tokyo, Japan) and incubated aerobically and anaerobically for 48 h. After incubation, colonies were counted and expressed as log_10_ CFU/slab.

### Quantification of biofilm-forming cells by real-time PCR

To quantify the number of biofilm-forming cells, real-time PCR was performed as previously described^57^. Briefly, bacterial genomic DNA was extracted from each biofilm sample using a DNA extraction kit (DNeasy PowerSoil Kit; Qiagen, Hilden, Germany) according to the manufacturer’s instructions and stored at − 20 °C until further analysis. The assays (20 μL) were prepared by mixing 1 μL of extracted DNA, 10 μL of SYBR Select Master Mix (Applied Biosystems, Carlsbad, CA, USA), 0.5 μL each of bacterial universal primers 27F (5′- AGRGTTTGATCMTGGCTCAG -3′) and 338R (5′- TGCTGCCTCCCGTAGGAGT -3′). Real-time PCR (Applied Biosystems™ 7500 Fast Real-Time PCR System; Thermo Fisher Scientific, Tokyo, Japan) was performed, and standard curves were generated using *S. mutans* ATCC 25175 genomic DNA to amplify serial dilutions. The experiments were repeated three times per sample. Data were acquired and analyzed using the software provided by the manufacturer (Applied Biosystems 7500 System SDS software version 2.0.2; Thermo Fisher Scientific, Tokyo, Japan).

### Confocal microscopy

The dentin slabs were washed in distilled water and stained using the LIVE/DEAD® Bacterial Viability Kit (BacLight™; Invitrogen, Carlsbad, CA, USA) for 30 min in the dark at room temperature. Thereafter, the slabs were observed using a confocal laser scanning microscope (LSM700; Carl Zeiss, Oberkochem, Germany), and five areas on the slabs were randomly selected and captured. The images obtained were reconstructed using an image analysis software (Imaris 9.2.1; Bitplane, Zurich, Switzerland), and the thickness of the biofilm as well as the volume of the dead and the live bacteria were assessed.

### SEM observation

Biofilm samples were prepared according to a previously described protocol^58^. The samples were immersed in 50% Karnovsky’s solution for 30 min before they were dehydrated in a series of aqueous ethanol solutions (50, 70, 80, 90, 95, and 100%) and transferred into *t*-butyl alcohol. Thereafter, the specimens were freeze-dried (JFD-320; JEOL, Tokyo, Japan) and sputter-coated with platinum (Sputter Coater SC7620; Quorum Technologies, East Sussex, UK) before observation under an SEM (JSM-6390LV; JEOL, Tokyo, Japan) using the secondary electron emission mode and at an accelerating voltage of 20 kV. Magnification of 7500 was used.

### 16S rRNA gene amplicon sequencing

PCR amplification targeting the V1–V2 hypervariable region of the bacterial 16S rRNA was performed separately using forward and reverse primers containing the adapter sequence with barcode index; 27F (5′-ACACTCTTTCCCTACACGACGCTCTTCCG ATCT-NNNNN-AGRGTTTGATYMTGG.CTCAG-3′) and 338R (5′-GTGACTGGA GTTCAGACGTGTGCTCTTCCGATCT-NNNNN-TGCTGCCTCCCGTAGGAGT-3′). Paired-end sequencing (2 × 300 bp) of the amplicons was performed using an Illumina MiSeq platform (Illumina, San Diego, CA, USA) according to standard protocols. Further, raw sequences obtained from all samples were de-multiplexed using the Fastq barcode splitter tool from the Fastx toolkit (version 0.0.13). Only the sequences that exactly matched the start of the reading sequence or primer were extracted. After removing the primer sequence, the QIIME pipeline (version 2.0) was applied to denoise and cluster quality-checked sequences into operational taxonomic units (OTUs) at a similarity cut-off of 97% using UCLUST (version 1.2.22q). Representative sequences for each OTU were aligned using the Ribosomal Database Project Classifier (http://rdp.cme.msu.edu/) against the Greengenes database, and each sequence was assigned to the genus level. The relative abundances of the taxa and the alpha and beta diversities were computed using the QIIME script (version 2.0).

### Statistical analysis

Statistical analyses were computed in R (RStudio version 1.2.1335; RStudio, Boston, MA, USA) for the Mac OS X development environment. Using the Shapiro–Wilk test, all sets of data were tested for normal distribution. The Friedman test was used to compare the mean CFU counts in aerobic and anaerobic conditions, including real-time PCR bacterial loads between groups and across days, followed by the Wilcoxon signed-rank test. Repeated measures ANOVA and multiple pairwise comparisons using Bonferroni’s correction were performed to compare the means of biofilm thickness in both groups and over different days. Further, dead and live cell volumes were calculated as ratios before analysis by the Friedman test and the Wilcoxon signed-rank test with Bonferroni’s correction. Based on the 16S rRNA sequencing data, Phyloseq and DESeq2 packages were used to perform bacterial community analysis^59,60^. For the alpha diversity index, Chao1 and Shannon indices, indicating taxa richness and abundance plus evenness, respectively, were determined using rarefied OTU tables. The Wilcoxon rank-sum test was used to compare the alpha diversity indices corresponding to each experiment day's control and SDF groups. Additionally, the Bray–Curtis distance matrix, a beta-diversity index, was measured from raw sequences that had been normalized based on the DESeq2 calculation. Using SHAMAN, a web application for metagenomic analysis^61^, a principal coordinates analysis (PCoA) plot was generated and a PERMANOVA test, which demonstrates the compositional difference across the groups of data, was performed. Comparative analysis of bacterial abundance was also performed using the DESeq2 method with a negative binomial model. The results were quantified and visualized as log2 fold change. Statistical differences (p-values) were corrected for multiple comparisons using the Benjamin-Hochberg method. For all tests, p-values < 0.05 were considered statistically significant.

## Supplementary Information

Supplementary Information.

## Data Availability

The data underlying this article cannot be shared publicly due to ethical concerns (the contents of agreements of the study). The data will be shared on reasonable request to the corresponding author.
