# Supplementary material for: An extensive description of the microbiological effects of silver diamine fluoride on dental biofilms using an oral in situ model
Source: Sci Rep. 2022 May 6;12:7435. doi: 10.1038/s41598-022-11477-1 (PMC9076617; doi:10.1038/s41598-022-11477-1)

**An extensive description of the microbiological effects of silver diamine fluoride on dental biofilms using an oral *in situ* model**

Supplementary Figures

Figure S1. A dentin slab-holding oral device utilised in this study. Each piece of device has eight rectangular slots for dentin slabs to be positioned. A 1×3 mm perforation was made to enable the growth of biofilm.


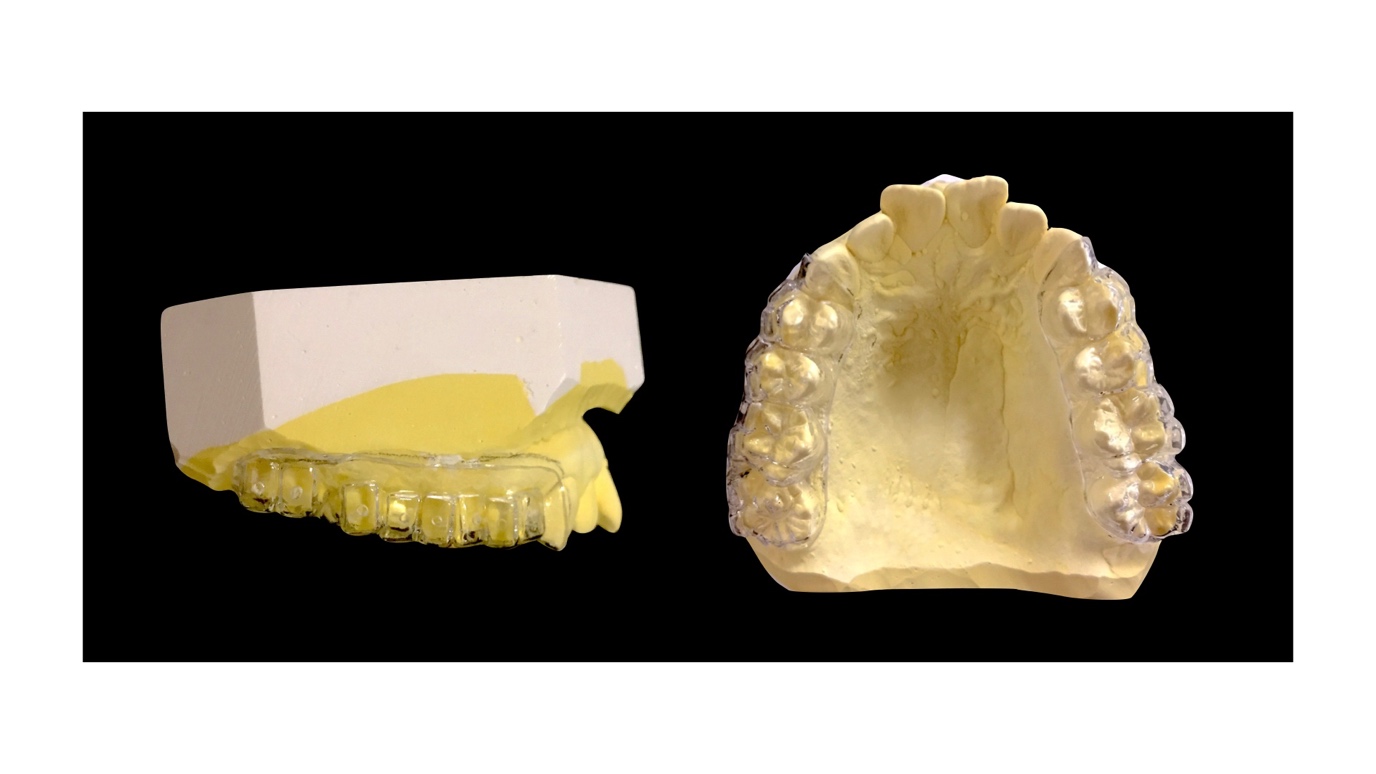


Figure S2. Dentin slab preparation protocol for control and SDF groups.


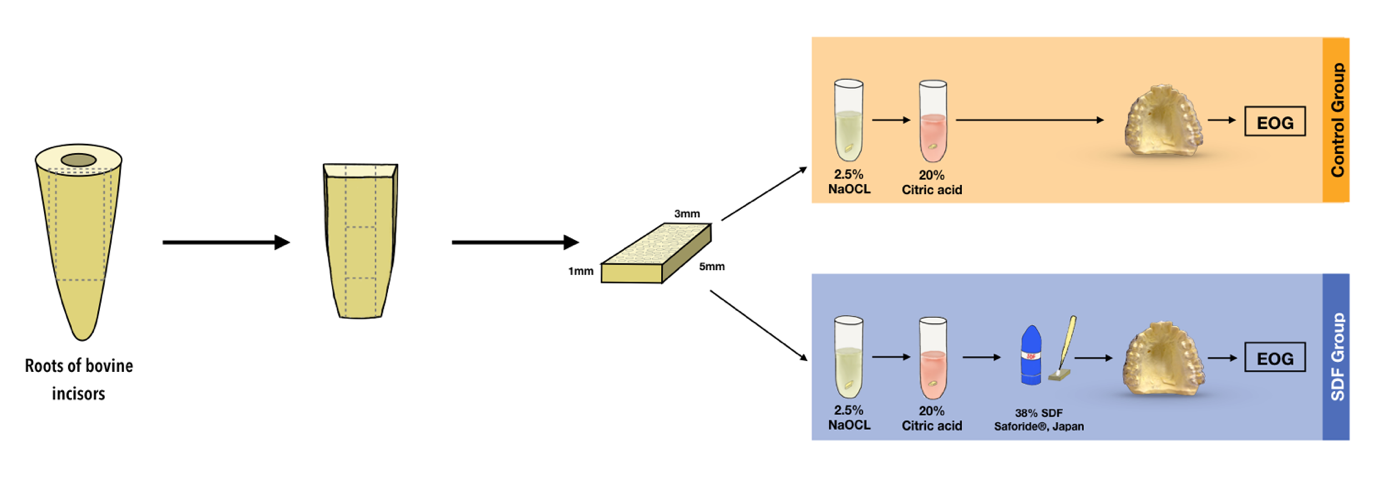

Supplement: Supplementary file 1 — Supplementary Information. [file 41598_2022_11477_MOESM1_ESM.docx]
